# Ultrasonographic renal sizes, cortical thickness and volume in Nigerian children with acute falciparum malaria

**DOI:** 10.1186/1475-2875-12-92

**Published:** 2013-03-13

**Authors:** Omolola M Atalabi, Adebola E Orimadegun, Ademola J Adekanmi, Olusegun O Akinyinka

**Affiliations:** 1 Department of Radiology, College of Medicine, University of Ibadan, Ibadan, Nigeria; 2 Institute of Child Health, College of Medicine, University of Ibadan, Ibadan, Nigeria; 3 Department of Paediatrics, College of Medicine, University of Ibadan, Ibadan, Nigeria

**Keywords:** *Falciparum* malaria, Renal sizes, Renal volume, Ultrasound

## Abstract

**Background:**

Utility of sonographic assessments of renal changes during malaria illness are rarely reported in African children in spite of the high burden of malarial-related kidney damage.

**Methods:**

In this case–control study, renal sizes, cortical thickness and volume of the kidneys of 131 healthy children and 170 with acute falciparum malaria comprising 85 uncomplicated malaria (UM) and 85 complicated malaria (CM) cases, measured within 24 hours of presenting in the hospital were compared.

**Results:**

The mean age of children with UM, CM and control groups was 49.7 ± 26.2 months, 50.7 ± 29.3 months and 73.4 ± 25.5 months, respectively (p < 0.001). The mean right kidney length of CM group was higher than control by 0.41cm (95% CI = 0.16, 0.65; p < 0.001) and UM by 0.32 cm (95% CI = 0.02, 0.62; p = 0.030). Similarly, mean left kidney length of CM was higher than control and UM by 0.34 cm (95% CI = 0.09, 0.60; p = 0.005) and 0.41cm (95% CI = 0.09, 0.72; p = 0.006), respectively. Estimated mean renal volume of the CM group was significantly higher than control group by 7.82 cm^3^ for right and by 5.79 cm^3^ for left kidneys respectively; in the UM group by 9.31cm^3^ for right and 8.87 cm^3^ for left kidneys respectively.

**Conclusion:**

There was a marginal increase in renal size of children with *Plasmodium falciparum* infection, which worsened with increasing severity of malaria morbidity. Ultrasonography provides important information for detecting renal changes in children with acute malaria.

## Background

Malaria remains a public health problem with significant morbidity and mortality posing major economic and developmental challenges in sub-Saharan Africa [1]. Malarial illness may take a variety of clinical forms, differing in pattern and severity, from uncomplicated to severe malaria. Despite efforts at controlling malaria, two out of ten children admitted to children emergency units suffer from severe forms of malaria and/or its complications [2]. Worldwide, about 90% of the reported cases and 85% of the deaths have been attributed to malaria in sub-Saharan Africa [3], where *Plasmodium falciparum* infection is responsible for almost all the morbidity and mortality.

The pathophysiology of severe falciparum malaria is complex and multifactorial with parasitized red blood cell destruction resulting in the release of haemoglobin and other toxic metabolites, up-regulation of cytokines, acute phase reactants all playing important pathogenic roles which may cause inflammation, tubulo-interstitial damage, glomerulonephritis and pigment nephropathy, all of which may lead to acute kidney injury (AKI) [4]. Moreover, previous studies [5,6] have demonstrated that cardiac output in children with severe malaria is adversely altered, but it is not clear from literature to what extent this alteration affects the kidneys. Data from Ghana [7] and Kenya [8] indicated that signs of shock, such as capillary refill, are common in children suffering from severe malaria. These adverse effects, though secondary rather than primary, support potential reversible ischaemic damage to the kidneys during acute malarial illness. Repeated *P*. *falciparum* infections can also result in nephron loss leading to chronic renal disease, including nephrotic syndrome, often non-responsive to steroid treatment [9-12].

Though the occurrence of haemoglobinuria and AKI is a significant complication in children with malaria, only a few studies have reported the magnitude. A recent study reported that as many as 19.1% of children with acute falciparum malaria developed haemoglobinuria [13]. AKI has been attributed to malaria in 13.7% [14] and 46.2% [15] in Nigeria. Weber *et al*. [16] in The Gambia observed that 25% of the cerebral malaria cases and 4% of children with mild malaria had AKI. Mortality from malaria-related AKI could be as high as 23% in endemic area [17].

Recent studies have shown that cases of severe malaria and its complications are on the increase probably because of the emergence of drug resistant parasites [18,19]. Early detection and monitoring of effects of malaria on the kidneys are important because timely interventions may prevent progression to irreversible damage. However, this is often very challenging in Africa mainly because of inadequate laboratory facilities. Currently, decisions relating to care of children with acute malaria are mostly guided by physical and biochemistry findings. Apart from the exorbitant cost of biochemistry tests, the turn-around times for getting reports from the laboratory often cause delay in treatments where available. Though ultrasound equipment may be available, sonological assessment of the kidneys in children with malaria is not routinely done, despite the fact that studies have validated the use of ultrasound in assessing renal functions in both clinical and epidemiological studies [20-22]. While other modalities can be used to determine kidney volume [23,24], ultrasound is preferred in most resource poor settings because it is relatively affordable and non-invasive. It is however, not known whether any change in renal sizes could be detected by using ultrasonographic scanning. This study, therefore, compared the length, width, anterio-posterior diameter and cortical thickness determined using ultrasound as well as estimated volume of kidneys in children with complicated and uncomplicated malaria with those of healthy children with no malaria parasitaemia.

## Methods

### Study design and setting

The study was case–control in design. Cases were recruited from the Children’s Clinics and Emergency Unit of the University College Hospital (UCH), Ibadan, Nigeria, while controls were selected among children living in the same neighbourhood of the respective cases. The UCH is a foremost tertiary referral hospital located in Ibadan, South-West of Nigeria. The yearly admissions in the Department of Paediatrics, UCH are approximately 2,500 with about 11% of them being cases of complicated malaria. Based on the 2006 National Census, Ibadan has an estimated population of 2,550,393. Children less than 15 years constitute about 19% of the population of Ibadan.

### Study population and sampling

Cases were children who presented with symptoms, signs and positive blood smear for malaria parasite and control were children of similar socioeconomic status who had no symptoms and negative blood smear for malaria parasite as control. Cases were grouped into uncomplicated and complicated malaria as defined by the World Health Organization [25]. Only eight children whose caregivers declined consent were excluded, but they received standard treatment according to the National guidelines on treatment of malaria.

### Sample size and power calculation

At the design stage of the study, it was assumed that utilizing ultrasound, there would be a mean difference of 13.0 cm^3^ in renal volume (this value was obtained from a pilot of 10 children) between cases and control. Therefore, studying unmatched 170 cases and 131 controls gave a statistical power (1-β) of over 90% at 95% confidence interval (CI).

### Data collection and laboratory procedures

Trained research Nurses and assistants administered a pre-tested structured record form to parents and their children at the time of recruitment. Each child was examined by the paediatrician with socio-demographic data, weight and height recorded. Laboratory investigations carried out included blood smear for malaria parasite counts, plasma urea and creatinine. The malaria parasites were counted against 200 white blood cells (WBC) and parasite density was calculated for each patient based on an assumed total WBC of 8,000/μL of blood [26]. All children with positive blood smear for malaria parasite were treated according to the Nigeria national anti-malarial treatment guidelines. Haemoglobin type of all participants were determined using gel electrophoresis method [27].

### Ultrasonographic procedures

All subjects had both kidneys scanned within 24 hours of presenting in the hospital using a portable Micromax Sonosite Inc. Bothell, WA, USA, with 5–8 MHz curved array transducer. All participants were scanned in the supine and decubitus positions in the longitudinal and transverse planes for length, width, anterio-posterior diameter and cortical thickness measured in centimeters. The liver and spleen were used as acoustic windows for the kidneys on the right and left respectively. The acquired measurements were recorded in a data form. Two certified sonologists performed the scan independently at each visit. These sonologists were blinded to the laboratory test results. The degree of agreement between findings reported by the sonologists was evaluated with the initial ten patients scanned (k = 0.9) in the pilot study.

### Variables, data handling and analysis plan

Data on kidney length, width, anterio-posterior size and cortical thickness were analysed using SPSS 16.0 statistical software (SPSS Inc. IL., USA). Kidney volume was estimated using the ellipsoid formula [23,28]. Renal sizes are known to vary with age and height [29], and since the three groups (control, UM and CM) differed significantly in age, ANCOVA was used to compare mean values of renal sizes adjusting for age. The Bonferroni procedure was adopted to manage Type I error that arose from multiple comparisons.

### Ethical considerations

Participation in the study was completely voluntary and based on written informed consent from child caregivers and assent of the children. Parents were made to understand that they were free to withdraw their consent at any time, and that they will continue to receive standard level of care even in such situation. Privacy of participants was maintained by using serial numbers on the case record forms. The study protocol was approved by the University of Ibadan/University College Hospital Ethical Review Committee.

## Results

### Clinical data

One hundred and seventy children with confirmed *P*. *falciparum* malaria comprising 85 uncomplicated and 85 complicated malaria cases and 131 healthy children with negative blood smear for malaria parasite participated in this study. There were more male than female children with malaria illness (M: F = 1.4: 1); (Table 1). The mean ages of children with UM (49.7 ± 26.2 months), CM (50.7 ± 29.3 months) and control group (73.4 ± 25.5 months) were significantly different (p = 0.001); (Table 1). The mean weight of control (14.6 ± 5.2 kg), UM (14.6 ± 4.1 kg) and CM (15.1 ± 5.4 kg) were not different (p = 0.056). Also, there were no significant differences in the mean heights of the three groups (Table 1). Only three had history of passing dark urine during the malarial illness and they all had positive results for blood using urine dipstick test. Other features of severe malaria documented among the CM group included: altered level of consciousness (n = 27), severe anaemia (n = 42), multiple convulsions (n = 7) and respiratory distress plus acidosis (n = 9). None of the study participants had signs of shock. Five children among the CM group also presented with oliguria (urine output <1.0 ml/m^2^/hour) which resolved within the first 24 – 36 hours of admission following administration of intravenous fluid in addition to other treatments. All the 42 children who had severe anaemia (haemoglobin <5 g/dl) were transfused with packed red blood cells at 12 to 15 ml per body weight (kg). However, no study participants had clinical signs of dehydration at the time of ultrasonographic scanning. All the four deaths recoded in this study had complicated (cerebral) malaria giving case fatality rate of 4.7% (4/85).

**Table 1:** Socio-demographic, clinical and laboratory parameters of children with malaria and controls

	**Control (131)**	**UM (85)**	**CM (85)**	**P**
Sex				
Male, n (%)	56 (42.7)	48 (56.5)	59 (69.4)	0.001
Female	75 (57.3)	37 (43.5)	26 (30.6)	
Age (months)				
Min – Max	26 – 120	26 – 108	24 – 108	
Median	72.0	48.0	48.0	
Mean	73.4 ± 25.5	49.7 ± 26.2	50.7 ± 29.3	0.001
Weight (kg)				
Min – Max	11.0 – 32.0	11.0 – 28.0	11.0 – 42.0	
Median	14.0	13.4	14.0	
Mean	14.6 ± 5.2	14.6 ± 4.1	15.1 ± 5.4	0.056
Height (cm)				
Min – Max	82.0 – 137.0	71.0 – 130.0	71.0 – 157.0	
Median	112.0	92.0	98.0	
Mean	111.5 ± 13.7	98.4 ± 18.2	94.9 ± 13.9	0.053
Malaria parasite counts (per μL)				
Min – Max	0	12,400 – 158,900	98,560 – 280,780	
Median	0	16,100	132,400	
Mean	0	19,306 ± 10,340	136,300 ± 97,500	<0.001
Plasma urea (mmol/L)				
Min – Max	4.8 – 14.6	3.6 – 25.7	3.6 – 43.9	
Median	6.2	6.6	11.9	
Mean	6.5 ± 2.2	6.8 ± 2.8	12.1 ± 9.0	<0.001
Plasma creatinine (mmol/L)				
Min – Max	17.7 – 86.1	18.8 – 88.4	18.8 – 88.4	
Median	53.0	44.0	53.0	
Mean	44.2 ± 8.8	53.0 ± 17.7	61.9 ± 53.0	<0.001

*Mann–Whitney U test used.

Normal values: Urea = 5 – 16 mmol/L, Creatinine = <88.8 mmol/L.

### Laboratory data

Mean values of malaria parasite counts for UM and CM were as shown in Table 1 with the CM group having a significantly higher counts than UM. Children in the control group had no malaria parasitaemia. The mean values of serum urea of children with CM (12.1 ± 9.0 mmol/L) was significantly higher than healthy control (6.5 ± 2.2 mmol/L) and UM (6.8 ± 2.8 mmol/L) groups (p < 0.001). Mean values of serum creatinine of the control (44.2 ± 8.8 mmol/L), UM (53.0 ± 17.7 mmol/L) and CM (61.9 ± 53.0 mmol/L) were significantly different with that of the CM group being the highest (Table 1). Of all the children who took part in the study, only 5 of those in the CM group had serum creatinine > 88.4 mmol/L. However, the mean renal volume of those 5 children with creatinine > 88.4 mmol/L was significantly higher than their counterparts in the same CM group who had normal creatinine (<88.4 mmol/L) by mean differences of 27.9 cm^3^ for right kidney and of 18.2 cm^3^ for the left. All the study participants had haemoglobin AA type.

### Renal ultrasound findings

The mean renal length, cortical thickness and estimated volume were compared between the right and left kidneys (Table 2). The age adjusted mean renal length of the left kidney in the control, UM and CM groups were significantly longer than the right in the corresponding groups (Table 2). The age adjusted mean renal width values in the control, UM and CM groups were slightly larger on the right than left but these differences were not statistically significant in all groups (Table 2). The adjusted mean A-P diameters however were not significantly different on both sides in control and UM groups but the right kidney had significantly higher A-P diameter of 4.05 cm compared to the left A-P diameter of 3.8 cm than left in the CM group (p < 0.001). The mean cortical thickness was significantly higher on the left than right in the UM and CM groups. No significant difference was however found between the adjusted renal volumes of the right and left kidney in all groups.

**Table 2:** Comparisons of kidney sizes, cortical thickness and estimated volume for children with malaria and controls

	**Right kidney**		**Left kidney**		**p**
	**Unadjusted mean**	**Adjusted mean**	**Unadjusted mean**	**Adjusted mean**	
Length (cm)					
Control	7.39 ± 0.89	7.13	7.85 ± 0.85	7.54	<0.001
UM	6.93 ± 1.01	7.21	7.20 ± 1.04	7.48	<0.001
CM	7.29 ± 1.13	7.54	7.63 ± 1.25	7.88	<0.001
Width (cm)					
Control	3.79 ± 0.57	3.62	3.71 ± 0.56	3.55	0.077
UM	3.29 ± 0.56	3.42	3.23 ± 0.56	3.35	0.118
CM	3.52 ± 0.67	3.65	3.48 ± 0.63	3.57	0.484
A-P diameter (cm)					
Control	3.93 ± 0.51	3.82	3.87 ± 0.49	3.77	0.196
UM	3.72 ± 0.69	3.88	3.65 ± 0.62	3.76	0.149
CM	4.01 ± 0.60	4.05	3.81 ± 0.48	3.86	<0.001
Cortical thickness (cm)					
Control	0.92 ± 0.18	0.89	0.96 ± 0.15	0.92	0.059
UM	0.81 ± 0.14	0.83	0.85 ± 0.16	0.87	0.006
CM	0.81 ± 0.13	0.83	0.85 ± 0.15	0.87	0.007
Estimated volume					
Control	59.27 ± 18.13	53.17	60.94 ± 16.52	55.22	0.083
UM	46.20 ± 18.70	51.68	47.28 ± 19.86	52.14	0.311
CM	56.01 ± 23.41	60.98	56.64 ± 25.05	61.01	0.967

UM = Uncomplicated Malaria, CM = Complicated malaria, AP = Anterio-posterior.

The estimated and adjusted mean values of kidney size, cortical thickness and renal volume are shown in Table 3. After adjusting for age, the right kidney length of CM group was significantly longer than control by 0.41 cm (95% CI = 0.16, 0.65; p <0.001) and UM by 0.32 cm (95% CI = 0.02, 0.62; p = 0.030) while there were no significant differences in left kidney length of control compared with UM and CM. Similarly, the mean left kidney length of CM was higher by 0.34cm (95% CI = 0.09, 0.60; p = 0.005) and 0.41 cm (95% CI = 0.09, 0.72; p = 0.006) for control and UM respectively. After adjusting for age, the mean width of the right kidney of control was significantly higher than UM by 0.20 cm (95% CI = 0.03, 0.36; p = 0.015), but not different from that of CM group. Also, the mean width of the kidney of CM was significantly wider than those of UM group in both right and left kidneys by 0.23 (95% CI = 0.03, 0.43; p = 0.018) and 0.22 (95% CI = 0.01, 0.43; p = 0.032) respectively. Conversely, comparisons of the mean AP diameters of kidneys of control, UM and CM showed no significant differences. While the control group had significantly higher mean cortical thickness than UM group (mean difference = 0.06cm for both kidneys), these values were significantly lower than those of CM in both right and left kidneys by 0.05cm (95% CI = 0.0, 0.11; p = 0.035) and 0.05 (95% CI = 002, 0.11; p = 0.037).

**Table 3:** Comparisons of kidney sizes, cortical thickness and estimated volume among children with malaria and controls

	**Right kidney**						**Left kidney**					
	**Unadjusted estimates**			**Adjusted estimates**			**Unadjusted estimates**			**Adjusted estimates**		
	**Mean diff**	**95% CI**	**p**	**Mean diff**	**95% CI**	**p**	**Mean diff**	**95% CI**	**p**	**Mean diff**	**95% CI**	**p**
Length												
Control vs. UM	0.46	0.19, 0.73	0.001	-0.08	-0.34, 0.17	1.000	0.65	0.37, 0.93	<0.001	0.06	-0.20, 0.32	1.000
CM vs Control.	0.29	-0.19, 0.37	0.516	0.41	0.16, 0.65	<0.001	0.23	-0.06, 0.51	0.121	0.34	0.09, 0.60	0.005
CM vs. UM	0.37	0.06, 0.67	0.019	0.32	0.02, 0.62	0.030	0.42	0.11, 0.74	0.008	0.41	0.09, 0.72	0.006
Width												
Control vs. UM	0.50	0.33, 0.66	<0.001	0.20	0.03, 0.36	0.015	0.48	0.32, 0.64	<0.001	0.20	0.03, 0.38	0.018
CM vs Control.	0.27	0.11, 0.44	0.001	0.03	-0.2, 0.13	1.000	0.23	0.07, 0.39	0.051	0.02	-0.15, 0.19	1.000
CM vs. UM	0.22	0.04, 0.41	0.017	0.23	0.03, 0.43	0.018	0.25	0.07, 0.42	0.007	0.22	0.01, 0.43	0.032
AP diameter (cm)												
Control vs. UM	0.21	0.05, 0.37	0.010	-0.07	-0.24, 0.11	1.000	0.22	0.08, 0.37	0.003	0.02	-0.15, 0.18	1.000
CM vs Control.	0.08	-0.08, 0.25	0.320	0.13	0.40, 0.51	0.511	0.07	-0.08, 0.21	0.383	0.09	-0.07, 0.25	0.540
CM vs. UM	0.30	0.12, 0.48	0.001	0.16	0.05, 0.37	0.181	0.16	0.00, 0.32	0.056	0.11	-0.09, 0.29	0.561
Cortical thickness (cm)												
Control vs. UM	0.12	0.07, 0.16	<0.001	0.06	0.01, 0.11	0.021	0.11	0.06, 0.15	<0.001	0.06	0.01, 0.11	0.032
CM vs Control.	0.11	0.07, 0.16	<0.001	0.05	0.0, 0.11	0.035	0.11	0.06, 0.15	<0.001	0.05	002, 0.11	0.037
CM vs. UM	0.00	-0.05, 0.05	0.920	0.07	0.06, 0.09	1.000	0.00	-0.05, 0.05	0.937	0.002	-0.06, 0.06	1.000
Estimated volume												
Control vs. UM	12.52	9.02, 17.10	<0.001	1.49	-3.48, 6.47	1.000	13.96	9.57, 18.34	<0.001	3.08	-2.27, 8.43	0.500
CM vs. Control	2.45	0.89, 7.41	0.388	7.82	2.91, 12.72	<0.001	5.13	0.61, 9.65	0.026	5.79	0.51, 11.07	0.026
CM vs. UM	9.81	5.27, 14.22	0.001	9.31	3.40, 15.22	0.001	8.82	3.89, 13.75	0.001	8.87	2.51, 15.24	0.003

Mean diff - Mean difference between groups; CI – Confidence Interval of Mean difference; adjustment was made for age.

UM = Uncomplicated Malaria, CM = Complicated Malaria, AP = Anterio-posterior.

The adjusted mean renal volume of control and UM were not significantly different. The adjusted mean renal volume of the CM group was significantly higher than control with mean differences of 7.82 cm^3^ for the right kidney (p <0.001) and 5.79 cm^3^ for left kidney (p = 0.026). Similarly, adjusted mean renal volume of the CM group was significantly higher than UM with mean differences of 9.31 cm^3^ for the right kidney (p = 0.001) and 8.87 cm^3^ for left kidney (p = 0.003).

In all increased parenchymal echogenicity of the kidneys were found in 12 children with complicated (14.1% of 85), eight (9.4%) had it in both kidney while four (4.7%) had it in the right kidney only. Increased parenchymal echogenicity of the kidneys was found in none of the control as well as those with uncomplicated malaria. There was no significant association between parasite counts and any of the kidney sizes. The mean renal length, cortical thickness and estimated volume of the three children who had dark coloured urine (haemoglobinuria) were not significantly different from others. Similarly, the mean values of renal sizes among four deaths were not significantly different from other children.

## Discussion

Many studies have reported the effects of malaria on the kidneys [25], with most describing biochemical indices as measure of malaria effects. The present study, it would appear, is the first to utilize ultrasound to assess renal sizes associated with acute falciparum malaria in Nigerian children. In this study, the average renal length of children with acute malaria whether uncomplicated or complicated was longer on the left than right while the left and right kidneys were similar in width and volume in the same individuals. Also, renal cortical thickness of the left kidney was slightly more than right in children with malaria. Though previous studies have shown that left and right kidneys were similar in sizes among healthy children except in length with the left being longer than right kidney in health and disease conditions [20,30,31]. It was difficult to get appropriate comparisons for the renal length in existing literature.

Moreover, the present study showed that UM group had increased length, width and cortical thickness in both right and left kidneys but not in the volume compared with healthy children. On the other hand, children with complicated malaria had increased renal lengths, width, and volume when compared with uncomplicated and control groups. These increased renal length, width, and volume were more in the CM group than UM. This finding suggests that the change in renal dimensions may have worsened with increasing severity of malaria illness. However, the relationship between renal length and volume has been controversially reported in previous studies. While Thakur *et al*. [32] in their study of 18 adult patients concluded that renal length as measured on CT scan is not a good predictor of renal volume in adult patients, some other authors recently reported good correlation between these two indices [23,33]. Widjaja *et al*. [33] reported that including parenchymal cortical thickness improved the prediction of renal volume. The increased cortical thickness in the UM and CM may have contributed to the overall renal volumes in children who participated in the present study.

Though the observed increased renal sizes in complicated malaria compared with mild malaria and healthy children were not surprising, the exact mechanism is not clear from our study. It is plausible that this observed difference in renal size may be due to the direct and indirect effects of severe manifestations of *P*. *falciparum* infection in the patients. Previous studies have shown that wide spread sequestration of parasitized red blood cells, excessive release of inflammatory cytokines and “sludging” effects of the parasite cause widespread swelling in many end-organs including the kidneys [25]. In the present study, there was no association between parasite counts and ultrasound measured renal sizes. This find is in line with the report by Dondorp and colleagues [34], who showed that peripheral parasitaemia is a poor reflection of whole body parasite mass.

One issue that restricts the generalization of findings from this study is the fact that cases and control were not effectively matched in terms of age and height. These two factors are important correlates of renal sizes. However, children who participated in the study had relatively comparable renal function as determined by the plasma creatinine and urea levels. Although both creatinine and urea levels are not reliable measures of renal function because they do not pick up subtle changes and there is also a significant time lag between onset of injury and elevation of plasma creatinine.

Magnetic Resonance Imaging and Computed Tomography have been shown to give better measures of renal length and volume using the disc-summation method [24], but in resource poor countries, these equipment are limited to very few tertiary health institutions and where available the cost is prohibitive. Ultrasound will continue to play a major role in the assessment of kidney sizes especially in children. Findings from this study therefore provide the basis for use of ultrasonographic examinations in the diagnosis and follow-up of children suffering from malaria especially severe falciparum malaria. It may aid in early detection and improve prognosis of renal complications associated with malarial illness. Considering the fact that ultrasound is cheap, widely available, and devoid of ionizing radiation, its use in the evaluation of children with acute falciparum malaria may be recommended. It is likely that the use of ultrasound may provide baseline information for further assessments of the kidneys during follow up of severe malaria in children in endemic areas.

## Competing interests

The authors declare that they have no competing interests.

## Authors’ contributions

AEO, OMA and OOA designed the research, AEO supervised the care of all children with malaria illness, OMA and AA performed the ultrasound scanning, and AEO, OMA, AA and OOA wrote the manuscript. All authors read and approve the final manuscript.
